# Immune Infiltration of CD8+ T Cells in Patients With Diabetic Pancreatic Cancer Reduces the Malignancy of Cancer Tissues: An *In Silico* Study

**DOI:** 10.3389/fendo.2021.826667

**Published:** 2022-01-25

**Authors:** Zheng Ye, Delin Liu, Dechen Liu, Yinqi Lv, Yidi Zhang, Jun Zhang, Jiantong Bao, Xuelu Yuan, Jiaying Hou, Ling Li

**Affiliations:** ^1^ Department of Endocrinology, Zhongda Hospital, School of Medicine, Southeast University, Nanjing, China; ^2^ State Key Laboratory of Bioelectronics, School of Biological Science and Medical Engineering, Southeast University, Nanjing, China; ^3^ Institute of Glucose and Lipid Metabolism, Southeast University, Nanjing, China; ^4^ Department of Clinical Science and Research, Zhongda Hospital, School of Medicine, Southeast University, Nanjing, China; ^5^ Department of Endocrinology, Yixing Second People’s Hospital, Wuxi, China; ^6^ Department of Endocrinology, Changji Branch, First Affiliated Hospital of Xinjiang Medical University, Xinjiang, China

**Keywords:** pancreatic cancer, diabetes, tumor microenvironment, miRNA-mRNA regulation network, immune therapy

## Abstract

**Background:**

Although the functional damage of the diabetic pancreas can affect the postoperative recovery of pancreatic cancer patients, there is no significant difference in the prognosis of pancreatic cancer patients with a history of diabetes and ordinary pancreatic cancer patients. There is still no practical theory to explain this phenomenon.

**Materials and Method:**

The mRNA expression profile data of 141 cases and 51 cases with clinical data of diabetes status were obtained from the TCGA database and the GEO database, respectively. The CRA001160 data set was obtained in the TISCH database. The Seurat was used to process single-cell expression profile sequencing data. The Cibersortx was used to construct a feature matrix of single-cell sequencing data and to deconvolve Bulk-RNAseq data to obtain each pancreatic cancer patients’ tumour invasion score. TIDE was used to assess the immune escape potential of the tumour. MiRNet was used to construct the miRNA-mRNA regulatory network.

**Result:**

Compared with regular pancreatic cancer patients, the immune-related signal transduction pathways in diabetic pancreatic cancer patients are in an activated state. In patients with diabetic pancreatic cancer, the infiltration score of CD8+ T cells is high, and the infiltration score of corresponding malignant tumour cells is low. The Bayesian classifier can distinguish diabetic pancreatic cancer patients from non-diabetic pancreatic cancer patients based on 10 signature genes. The miRNA-mRNA regulatory network suggests that regulation by miRNA can influence mRNA expression and thus prognostic survival of pancreatic cancer patients.

**Conclusion:**

The activation of inflammatory-related signalling pathways in diabetic pancreatic cancer patients increases the immune infiltration of CD8+ T cells in cancer patients and reduces the development of malignant tumour tissues. The expression of 10 signature genes allowed the diagnosis of diabetic and non-diabetic pancreatic cancer patients. The miRNA-mRNA regulatory network may be the main cause of the differences in the tumour inflammatory microenvironment between the two groups of patients. These findings help us further understand the immune microenvironment of patients with diabetic pancreatic cancer.

## Introduction

Pancreatic cancer has one of the highest mortality rates of any cancer type, with an overall five-year survival (OS) rate of less than 5%. Despite the tremendous breakthroughs in cancer treatments with advances in medical technology and complementary therapies, the prognosis for pancreatic cancer patients remains poor (1, 2). Understanding the pathogenesis of pancreatic cancer and the factors that drive its rapid growth is of great importance to the treatment and control of pancreatic cancer. Numerous studies have shown that diabetes is a major risk factor for the development of pancreatic cancer. Diabetes mellitus is a metabolic disease characterised by high blood sugar. Prolonged hyperglycemia causes chronic damage and dysfunction in various tissues, especially the eyes, kidneys, heart, blood vessels and nervous system. A large number of studies have reported an epidemiological association between diabetes and pancreatic cancer. Data have shown that approximately 50% of newly diagnosed patients with pancreatic cancer are diabetic (3, 4). The incidence of pancreatic cancer among new diabetics may reach 0.85%, which is eight times higher than expected. Despite the fact that diabetes affects the recovery of patients after radiotherapy, chemotherapy and surgical resection of pancreatic cancer, there is no significant difference in the overall survival of pancreatic cancer patients with diabetes (5, 6). Although this conclusion is still controversial, no well-established theory is still available to explain the phenomenon.

The tumour microenvironment of pancreatic cancer is of great importance to the progression and metastasis of the pancreas. The pancreatic cancer microenvironment is mainly composed of cancer cells, stromal cells and extracellular components. The main cells that promote the progression of pancreatic cancer are pancreatic stellate cells (PSC), regulatory T cells (Treg), myeloid suppressor cells (MDSC) and tumour-associated macrophages (TAM). These cells can co-maintain the microenvironment through the exocrine secretion of a number of cellular matrices and inflammatory factors (7). The main features of the pancreatic cancer microenvironment are hyperdense cell proliferation and extensive immunosuppression (8). The pancreatic cancer microenvironment promotes the proliferation of pancreatic cancer and escapes immune surveillance by directly suppressing tumour immunity and inducing the proliferation and metastasis of immunosuppressed cells. No research team has focused on the characteristics of the tumour microenvironment in diabetic pancreatic cancer patients. Widespread elevated levels of inflammatory factors are present in diabetic patients (9). The increased levels of these inflammatory factors may affect the tumour microenvironment in diabetic patients with pancreatic cancer, thereby triggering crosstalk of the cellular components of the pancreatic cancer microenvironment. A recent study has shown that a high-glucose environment in tumors can promote the proliferation of immune-related cells in tumors, without significantly affecting tumour cells. The main reason for the high rate of glucose consumption by tumors is not the cancer cells, but the immune cells in the tumour tissue. All these studies suggest a complex state of the tumour microenvironment in diabetic pancreatic cancer patients (10).

In this study we compared the differences in expression profiles between diabetic and non-diabetic pancreatic cancer patients by two separate data sets (TCGA PAAD, GSE79668). The composition of the 13 types of cells in the tumour microenvironment from these bulk-RNAseq data was assessed by a matrix of features of the 13 types of cells in the pancreatic cancer microenvironment obtained from single-cell sequencing data (CRA001160), revealing differences in the tumour microenvironment of diabetic versus non-diabetic pancreatic cancer. Differential miRNAs from diabetic and non-diabetic pancreatic cancer patients were compared by TCGA PAAD miRNA sequencing data, and miRNA-mRNA regulatory networks were constructed from differentially expressed genes. These results have important implications for our further understanding of the unique characteristics of the tumour microenvironment and the progression of pancreatic cancer in diabetic pancreatic cancer patients. On the other hand, it also reveals that CD8+ T cell-based immunotherapy may be effective in pancreatic cancer; interfering with the immune microenvironment of the tumour through miRNA may be an effective means to improve the prognosis of pancreatic cancer.

## Methods

### Patients and Datasets

The mRNA expression profile and microRNA expression profile data for 141 PAAD cancer samples were downloaded from the TCGA database (11) (HTTPS://portal.gdc.cancer.gov/projects/TCGA-HNSC) and included information on their clinicopathology. Among them were 35 patients with diabetic pancreatic cancer and 106 patients with non-diabetic pancreatic cancer. Fifty-one pancreatic cancer samples from GSE79668 (12) were downloaded from the GEO database (13), along with clinicopathological information on these samples. The numbers of patients with diabetic pancreatic cancer and non-diabetic pancreatic cancer were 22, 29 respectively. The single cell expression profile matrix for CRA001160 (14) was downloaded from TISCH (15) and contains expression profile data for 57,443 single cells. Cell annotation information from the data source paper was used in this study for a total of 13 major cell types including Acinar, B, CD8Tex, Ductal, Endocrine, Endothelial, Fibroblasts, M1, Malignant, Monocyte, pDC, Plasma, Stellate.

### Overexpression Analysis and GSEA

Differential analysis of mRNA expression profiles between diabetic pancreatic cancer and non-diabetic pancreatic cancer patients in PAAD samples was performed by the DESeq2 (16) package of R software (FDR<0.05, |log2foldchange|>1). Gene Ontology (GO) (17) and Kyoto Encyclopedia of Genes and Genomes (KEGG) (18) functional annotations were done for up- and down-regulated genes in diabetic pancreatic cancer by the ClusterprofileR (19) package of R software, respectively. The differentially expressed genes were ranked according to their log2foldchange and functional enrichment analysis was performed on the differentially expressed genes using GSEA (20).

### Assessment of Tumour Microenvironmental Status in Pancreatic Cancer

The pancreatic cancer tumour tissue single cell sequencing dataset (CRA001160) was non-linearly dimensionalised, clustered, visualized and annotated with 13 major cell types by the Seurat package of R software (21). One hundred cells from each major cell type were randomly selected to reconstitute a pancreatic cancer tumour microenvironment expression profile of 1300 cells. The CIBERSORTx (22) was used to process the new single cell expression profiles to construct a signature matrix of these 13 major cell types. Using these 13 cell type feature matrices, the 141 pancreatic cancer patients from TCGA PAAD and 51 pancreatic cancer patients from GSE79668 were deconvoluted respectively, resulting in an ABSOLUTE score matrix of the 13 major cell types in the tumour tissues of these pancreatic cancer patients. The higher the Absolute score for a cell type, the higher the absolute percentage of this cell type in the tumour tissue. The TIDE (http://tide.dfci.harvard.edu/faq/) (23) computational framework was used to assess the functional status of T lymphocytes in the tumour microenvironment. Using the TIDE computational framework, scores for tumour immune dysfunction and immune rejection status can be obtained. These scores could be applied to assess the potential for tumour immune escape.

### Construction of a Classifier for Distinguishing Pancreatic Diabetes Mellitus From Non-Pancreatic Diabetes Mellitus

In order to diagnose pancreatic cancer diabetes versus non-pancreatic cancer diabetes at the expression profile level, machine learning models were used to construct diagnostic models. Firstly, the voom function of the limma (24) package of the R software was used to transform the count matrix of the expression profile into a normalized expression profile matrix. The removebatcheffect function was then used to remove the batch effect from the TCGA PAAD dataset and the GSE79668 dataset. the TCGA PAAD dataset was used for feature extraction and machine learning model construction and the GSE79668 dataset was used to assess the generalization capability of the model. Four machine learning models (SVM, Random Forest, Naïve Bayes, Logistic Regression) were used to construct classifiers for diabetes and non-diabetes. Two metrics, ROC curve and Calibration curve, were used to evaluate the performance of the models.

### Construction of miRNA-mRNA Regulatory Network and Survival Analysis

Differential expression analysis of the count matrix of miRNA expression profiles of TCGA PAAD patients (diabetic pancreatic cancer vs. non-diabetic pancreatic cancer) was done by DESeq2 of R software. The miRNAs with significant differences were obtained according to the filtering conditions of pvalue<0.001,|log2foldchange|>0.5. Venn diagrams were used to visualize the relationship between differential genes in diabetic pancreatic cancer and non-diabetic pancreatic cancer in TCGA PAAD and GSE79668. Genes that were significantly upregulated in both datasets were used to construct miRNA-mRNA regulatory networks. The miRNet (25) (https://www.mirnet.ca/) was used to construct the miRNA-mRNAt regulatory network. miRNA target genes were predicted using the miRTarBase V8.0 database (26). The network nodes were pruned according to their degree, and nodes with degree > 1 were retained. The networks were then functionally annotated by GO, KEGG and REACTOME (27) databases. Finally Kaplan-Meier Plotter (28) (https://kmplot.com/analysis/) was used to do survival analysis on the screened differential miRNAs.

### Statistical Analysis

Absolute scores of cell types were compared between groups using the Wilcox test. Univariate survival analysis was performed by Kaplan-Meier survival analysis with the log-rank test. Orange3 (version:3.28) (29) was used to build machine learning models.

## Result

### Differential Expression Profiles of Tumor Tissues From Diabetic and Non-Diabetic Pancreatic Cancer Patients

**Figure 1:**
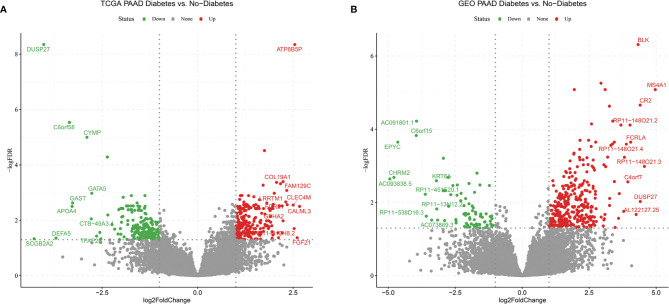
Volcano plot **(A)** Differentially expressed genes in the TCGA PAAD dataset for diabetic vs. non-diabetic. **(B)** Differentially expressed genes between diabetic and non-diabetic in the GSE79668 dataset. FDR<0.05,abs(log2FoldChange)>1 used as cutoff value for the volcano plot.

**Figure 2:**
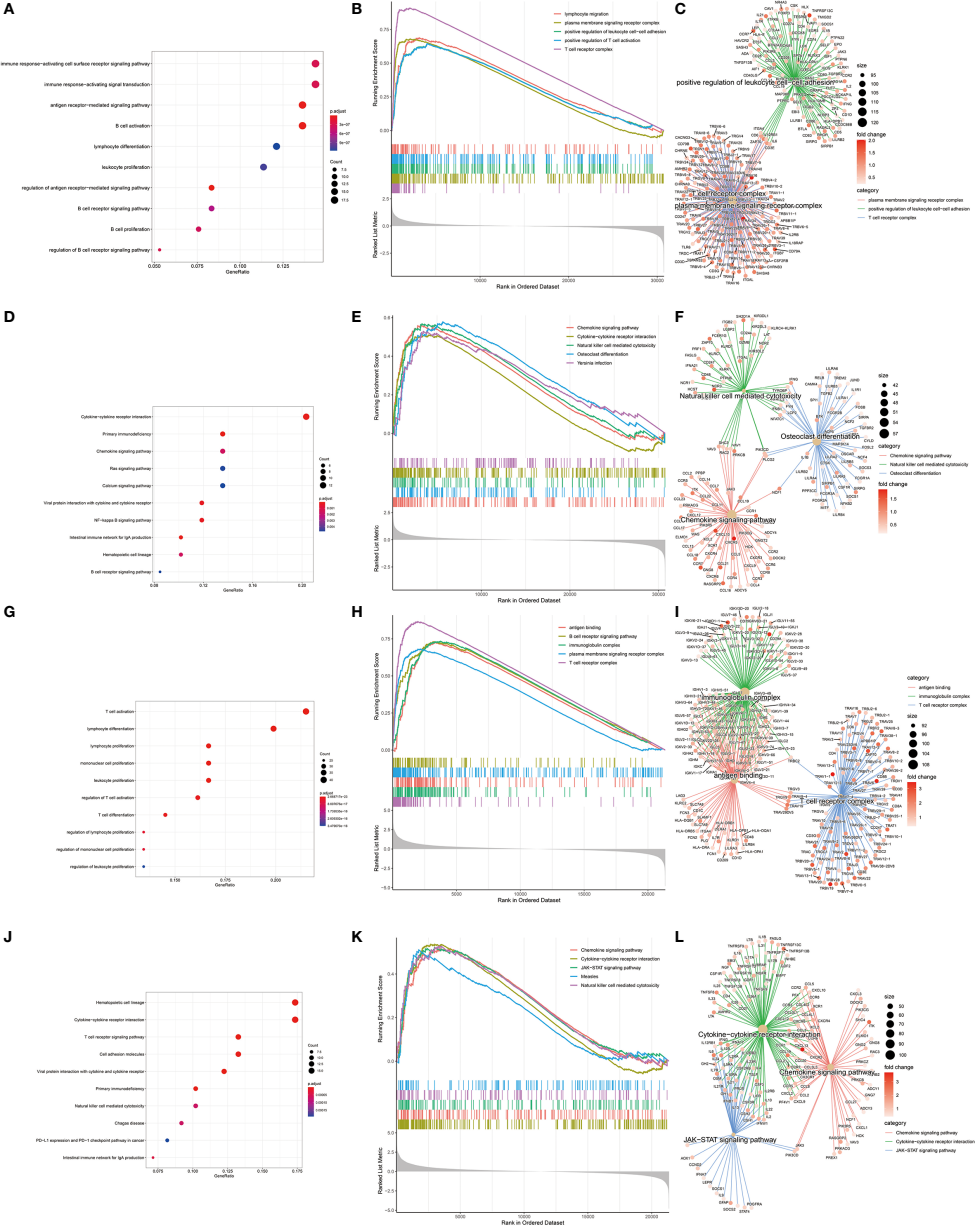
KEGG, GEO enrichment analysis of upregulated genes in diabetic pancreatic cancer patients and GSEA results. **(A–C)** Functional enrichment of GO overexpressed genes, GSEA and gene regulatory networks in the TCGA PAAD dataset, respectively. **(D–F)** Functional enrichment of KEGG overexpressed genes, GSEA and gene expression regulatory network in TCGA PAAD dataset, respectively. **(G–I)** Functional enrichment of GO overexpressed genes, GSEA and gene expression regulatory network in the GSE79668 dataset, respectively. **(J–L)** is the functional enrichment of KEGG overexpressed genes, GSEA and gene expression regulatory network in the GSE79668 dataset, respectively.

In order to find differences between diabetic and non-diabetic pancreatic cancer patients, DESeq2 was used to investigate the differences in expression profiles between the two groups. In the TCGA PAAD dataset, 215 genes were upregulated and 190 genes were downregulated in cancer tissues from patients with diabetic pancreatic cancer (**Figure 1A**). 338 genes were upregulated and 79 genes were downregulated in tumour tissues from patients with diabetic pancreatic cancer (**Figure 1B**) in the GSE79668 dataset (FDR<0.05, | log2Foldchange|>1). GO and KEGG databases performed functional enrichment analysis and GSEA for upregulated and downregulated genes in these two datasets, respectively. The functional enrichment results of the TCGA PAAD dataset showed that among the up-regulated genes, the overexpressed genes analysed by GO enrichment were mainly enriched in immune response-activating cell surface receptor signaling pathway, immune response -activating signal transduction, antigen receptor-mediated signaling pathway and other signaling pathways (**Figure 2A**). The GSEA results showed that the higher ranked genes in the GO database were mainly enriched in signalling pathways such as lymphocyte migration, plasma membrane signaling receptor complex, positive regulation of leucocyte cell-cell adhesion, positive regulation of T cell activation, T cell receptor complex (**Figures 2B, C**). In the KEGG database, these upregulated genes are mainly enriched in signalling pathways such as Cytokine-cytokine receptor interaction, Primary immunodeficiency, Chemokine signaling pathway (**Figure 2D**). The GSEA results showed that the higher ranked genes in the KEGG database were mainly enriched in signalling pathways such as Chemokine signaling pathway, Cytokine-cytokine receptor interaction, Natural killer cell mediated cytotoxicity, Osteoclast differentiation, Yersinia infection (**Figures 2E, F**). The functional enrichment of the GSE79668 dataset showed that the upregulated genes in the GO database were mainly enriched in signalling pathways such as T cell activation, lymphocyte differentiation, lymphocyte proliferation (**Figure 2G**). The results of GSEA enrichment showed that Antigen binding, B cell receptor signaling pathway, immunoglobulin complex, plasma membrance signaling receptor complex, T cell receptor complex were activated in the tumor tissues of pancreatic cancer and diabetes (**Figures 2H, I**). In the KEGG database, overexpressed genes were mainly enriched in signal pathways such as Hematopoietic cell lineage, Cytokine-cytokine receptor interaction, and T cell receptor signaling pathway (**Figure 2J**). The results of GSEA enrichment showed that Chemokien signaling pathway, Cytokine-cytokine receptor interaction, JAK-STAT signaling pathway, Measles, Natural killer cell mediated cytotoxicity and other signaling pathways were in an activated state (**Figures 2K, L**). In contrast, the two datasets showed relatively large differences in the results of signalling pathway enrichment in the genes that were down-regulated (**Supplementary Figure S1**). These results suggest that inflammatory and immune-related signalling pathways are extensively activated in the tumour tissue of diabetic pancreatic cancer patients. This “hot” immune state reflects the specific tumour microenvironment of tumour tissue in diabetic pancreatic cancer patients.

### Differences in the Tumour Microenvironment of Tumour Tissue in Diabetic and Non-Diabetic Pancreatic Cancer Patients

**Figure 3:**
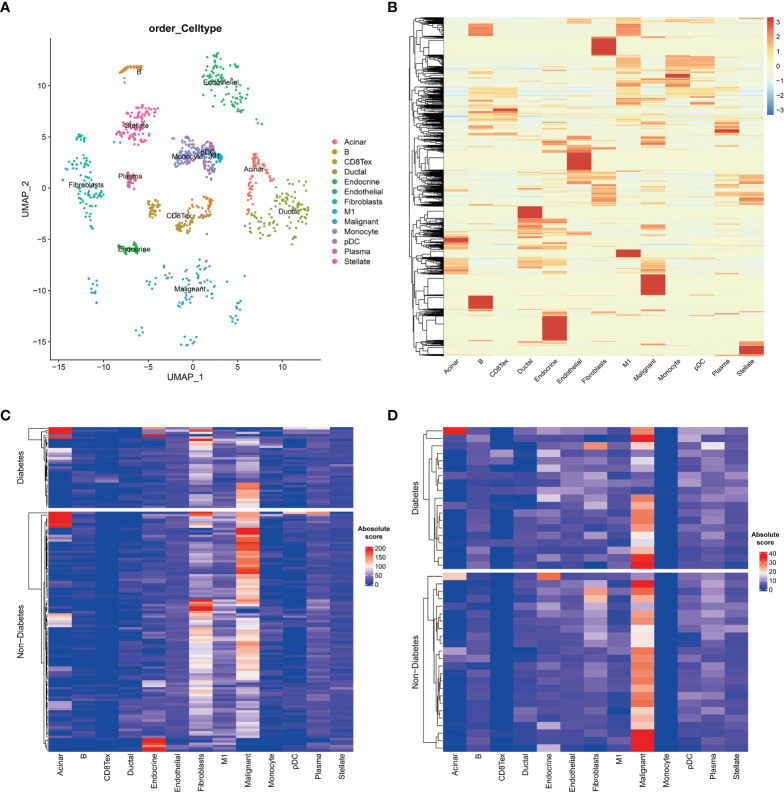
Cell type expression profile signature matrix. From the 13 clustered cell populations of the PAAD scRNAseq dataset, 100 cells of each cell type were randomly screened and the expression profile feature matrix of these cell types was obtained by the CIBERSORTX algorithm. **(A)** Two-dimensional scatter clustering plot of umap for the 13 cell types obtained by PAAD scRNAseq. **(B)** Feature matrices obtained by the CIBERSORTX algorithm. **(C)** Absolute score clustering heat map of the 13 cells from the TCGA PAAD dataset. **(D)** Absolute score clustering heat map of 13 cells from the GSE79668 dataset.

**Figure 4:**
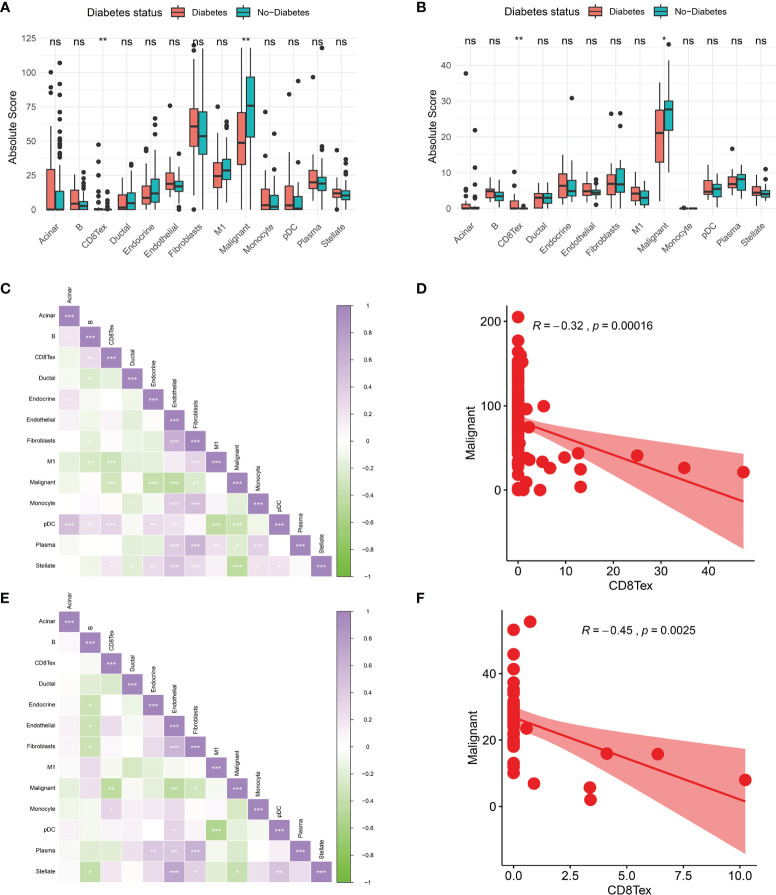
Comparison of the tumor immune microenvironment in patients with diabetic pancreatic cancer versus non-diabetic pancreatic cancer. **(A)** Mean value test of Absolute Score of tumor tissues of 13 cells in the TCGA PAAD dataset. **(B)** Mean value test (wilcox-test) of Absolute Score of tumour tissue from 13 cells in the GSE79668 dataset. **(C)** Correlation of Absolute scores of 13 cell types in the TCGA PAAD dataset. **(D)** Correlation of Malignant and CD8Tex in the TCGA PAAD dataset. **(E)** Correlation of Absolute scores of 13 cell species in the GSE79668 dataset. **(F)** Correlation between Malignant and CD8Tex in the GSE79668 dataset. (Spearman correlation test). ns, Not Significant, *p < 0.05, **p < 0.01, ***p < 0.001.

To further investigate the tumour microenvironment in diabetic patients with pancreatic cancer, the CRA001160 dataset was used to construct a signature matrix of the 13 major cell types in pancreatic cancer. We constructed a new expression profile matrix consisting of 1300 cells from 13 major cell types, 100 cells of each type were randomly selected (**Figure 3A**). The Cibersortx was used to construct a signature matrix of the pancreatic cancer tumour microenvironment. Ultimately, a feature matrix of 13 cell types, consisting of 3706 genes, was constructed (**Figure 3B**). Each cell type has its own unique expression pattern. The signature matrix was used to deconvolve the bulk-RNAseq matrix. We eventually obtained heat maps of the tumour microenvironment distribution for the TCGA PAAD dataset (**Figure 3C**) and the GSE79668 dataset (**Figure 3D**). As can be observed from the figure, the tumour immune microenvironment showed significant differences in both Diabetes and Non-Diabetes in the two independent datasets. In the TCGA PAAD dataset, CD8Tex was significantly higher in the Diabetic pancreatic cancer than in the Diabetic group (p<0.01), while the opposite was true for Malignant (p<0.01). Fibroblast and Malignant are the most predominant components of pancreatic cancer tissue (**Figure 4A**). In the GSE79668 dataset, we similarly found that CD8Tex immune infiltration was significantly higher in diabetic pancreatic cancer patients than in the non-diabetic group (p<0.01), while Malignant composition was significantly lower than in the non-diabetic group (p<0.05) (**Figure 4B**). It further suggests that the tumour microenvironment in pancreatic cancer diabetes is in a “hot” immune state and that malignant cell infiltration is significantly lower in this state. To further elucidate the characteristics of the immune microenvironment in pancreatic cancer tumour tissue, the Absolute Score of 13 cells was used to calculate the correlation of these cells. The results suggest that Malignant showed a significant negative correlation with Endocrine, Endothelial, Fibroblast, pDC, Plasma, and Stellate in the TCGA PAAD dataset (p<0.05). CD8Tex, on the other hand, showed a significant positive correlation (p<0.05) with B cells, pDC, and Stellate cells, and a negative correlation with M1 (**Figure 4C**), where CD8Tex and Malignant’s Absolute Score showed a significant negative correlation (R=-0.32, p<0.001) (**Figure 4D**). Malignant showed a negative correlation with Endocrine, Fibroblasts, Stellate in the GSE79668 dataset (p<0.05). CD8Tex showed a positive correlation (p<0.05) with Monocle (**Figure 4E**). In the GSE79668 dataset, CD8Tex and Malignant also showed a significant negative correlation (R=-0.45, p<0.01) (**Figure 4F**). These results suggest a relationship between cells in the unique tumour microenvironment of diabetic pancreatic cancer.

### Relationship Between Tumour Microenvironment and Prognosis of Pancreatic Cancer Patients

**Figure 5:**
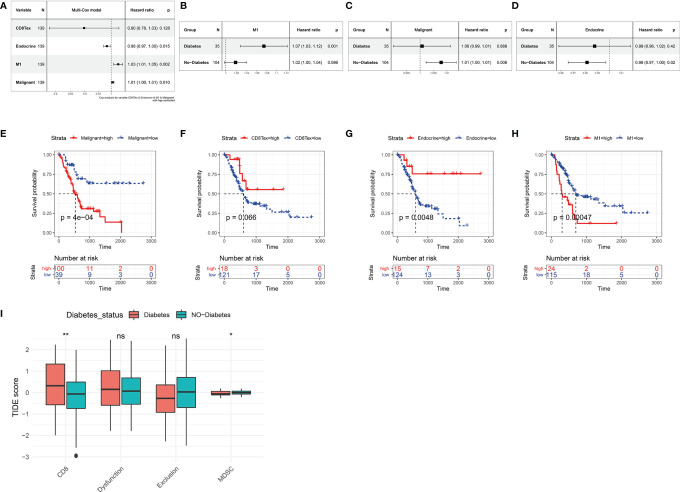
Relationship between tumor microenvironment components and prognostic survival of cancer patients in the TCGA PAAD dataset. **(A)** Relationship between the proportion of CD8Tex, Endocrine, M1, Malignant in the tumor microenvironment and the prognosis of PAAD patients. **(B)** Relationship between immune infiltration of M1 cells and prognostic survival in diabetic and non-diabetic pancreatic cancer patients. immune infiltration of M1 cells is a high risk factor in diabetic pancreatic cancer patients. **(C)** Relationship between infiltration of Malignant cells and prognostic survival in diabetic and non-diabetic pancreatic cancer patients. In non-diabetic pancreatic cancer, infiltration of Malignant is a high risk factor. **(D)** Association of Endocrine cell scoring in the tumour microenvironment with prognostic survival in patients with diabetic and non-diabetic pancreatic cancer. In patients with non-diabetic pancreatic cancer, the proportion of Endocrine was a beneficial factor. **(E–H)** The Absolute scores of Malignant, CD8Tex, Endocrine, and M1 were used to group PAAD patients and compare the differences in prognostic survival between patients in higher and lower groups, respectively. **(A–D)** All used one-way cox proportional regression models to assess HR for risk factors, logrank was used to test the statistical significance of the results, and all used the patient’s age as a correction factor. **(I)**TIDE computational framework to assess the immune functional status of the tumour microenvironment. ns, Not Significant, *p < 0.05, **p < 0.01, ***p < 0.001.

Based on the Absolute Score obtained by CibersortX, it is possible to assess the relationship between the cells that make up the tumour microenvironment of pancreatic cancer and the prognostic survival of the cancer. Immune infiltration of M1 in the TCGA PAAD dataset was then a high risk factor for pancreatic cancer (**Figure 5A**, HR=1.03, p<0.01), in both diabetic and non-diabetic groups (**Figure 5B**). Malignant was also a high risk factor for pancreatic cancer (**Figure 5C**, HR=1.01, p<0.05), however it showed no significance in the diabetic group. A higher Endocrine score was a beneficial factor for pancreatic cancer (**Figure 5A**, HR=0.98, p<0.05), a result that was the same in the non-diabetic group, yet showed greater individual variability in the diabetic group (**Figure 5D**). The results of the Kaplan-Miere survival analysis showed that patients with high CD8Tex and Endocrine scores showed better prognostic survival, while patients with high Malignant and M1 scores showed poorer prognostic survival (**Figures 5E–H**). However, in the GSE79668 dataset, the results of the survival analysis were not statistically significant due to the small number of samples (**Supplementary Figure S2**).

### Comparison of Immune Cell Function in the Immune Microenvironment of Diabetic Pancreatic Cancer and Non-Diabetic Pancreatic Cancer

In our previous study, we found a higher degree of immune infiltration of CD8Tex in the tumour microenvironment of diabetic pancreatic cancer patients compared to normal pancreatic cancer. To further investigate the functional status of these CD8Tex in pancreatic cancer diabetes, the TIDE calculation framework was used to assess the immune dysfunction and exclusion status of pancreatic cancer tumour tissue. We combined TCGA PAAD and GSE79668 to assess the tumour immune status of each sample using the TIDE calculation framework (**Figure 5I**). The results showed that the score of CD8 was higher in diabetic pancreatic cancer compared to non-diabetic pancreatic cancer (wilcox.test,p<0.001), while the score of Myeloid-derived suppressor cells was lower (wilcox.test,p<0.05). This result is consistent with the previous findings, suggesting that the tumour microenvironment in diabetic pancreatic cancer is in a “hot” immune state. DysFunction scores did not differ significantly between the two groups, indicating that the immune function of T lymphocytes in the tumour microenvironment of pancreatic cancer did not significantly diverge between the two subgroups. In addition, although Exclusion scores did not show a significant difference between the two subgroups, immune infiltration scores were lower in the diabetic pancreatic cancer group (p<0.1). The above findings suggest that diabetic pancreatic cancer patients have a higher degree of immune infiltration of T lymphocytes than non-diabetic patients, and that the immune function status of T lymphocytes may be even better.

### Construction of a Classifier for Diabetic and Non-Diabetic Pancreatic Cancer Patients

**Figure 6:**
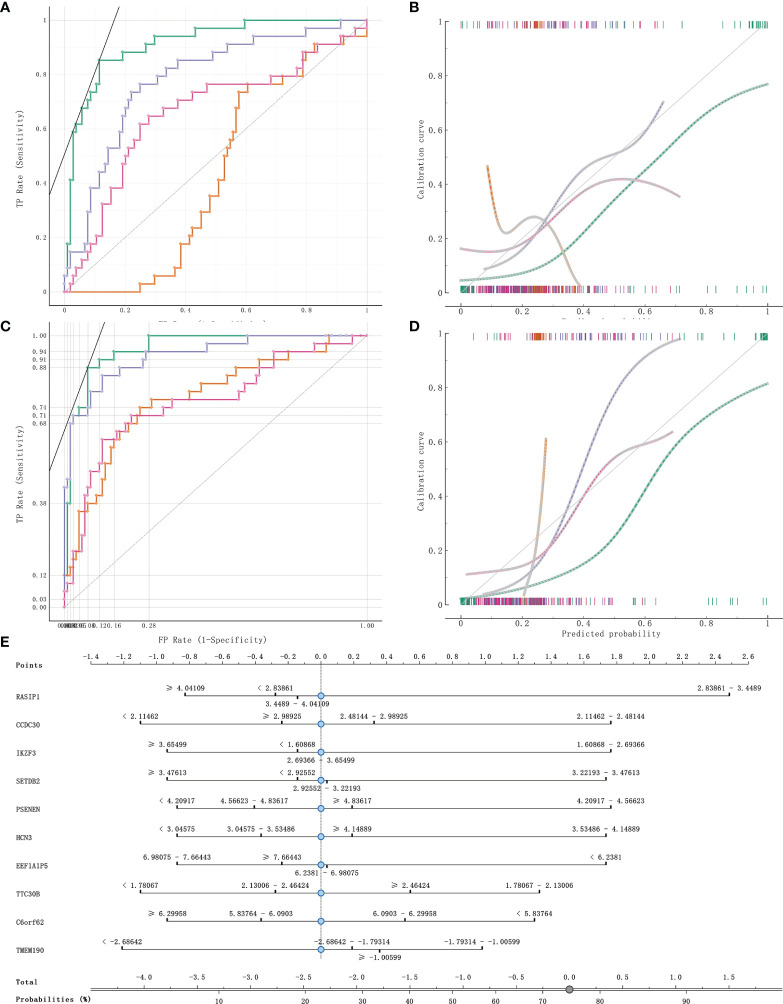
Model evaluation and model validation of classification models. **(A, B)** training dataset (TCGA PAAD sample set) used to evaluate AUC and model correction curves for 4 classifiers. **(C, D)** testing dataset (GSE79668) used to evaluate AUC and model correction curves for 4 classifiers. **(E)** The nomogram plot of the naïve Bayesian model. Green refers to Naïve Bayes, orange to SVM, cyan blue to RandomForest and rose to logistic regression.

141 cases from the TCGA PAAD dataset were used to train a machine learning classifier for differentiating pancreatic cancer diabetes from non-pancreatic cancer diabetes. Using Gini ratio, we obtained 10 signature genes from the TCGA PAAD dataset for differentiating pancreatic cancer diabetes from non-pancreatic cancer diabetes (**Table 1**). The 10 genes are RASIP1, CCDC30, TTC30B, PSENEN, IKZF3, SETDB2, HCN3, TMEM190, EEF1A1P5, C6orf62. Leave one out was used to assess the stability of four machine learning models. The AUC, CA, F1, Precision, Recall were used to assess the predictive power of the models. The results show that the Naïve Bayes classifier performs best in this binary classification task (**Table 2**, **Figures 6A, B**). In the validation set, the Naïve Bayes classifier also obtained the best performance (**Table 3** and **Figures 6C, D**). We constructed a Nomogram of the Naïve Bayes classifier based on these 10 features (**Figure 6E**). The main role of this model is to be used to classify the large number of samples with unlabelled diabetes status in the GEO database, thus helping the researcher to obtain a larger number of usable samples.

**Table 1:** Feature gene extraction.

Gene	Info. gain	Gain ratio	Gini	ANOVA	χ²	ReliefF	FCBF
RASIP1	0.135	0.067	0.049	4.037	3.577	0.042	0.106
CCDC30	0.127	0.064	0.059	3.837	1.761	0.028	0.100
TTC30B	0.114	0.057	0.056	4.196	5.255	0.013	0.000
PSENEN	0.107	0.054	0.047	3.150	1.360	0.035	0.000
IKZF3	0.106	0.053	0.047	6.338	4.898	0.025	0.082
SETDB2	0.104	0.052	0.046	4.631	1.181	0.051	0.000
HCN3	0.104	0.052	0.045	4.594	7.702	0.041	0.000
TMEM190	0.103	0.051	0.056	8.716	10.614	0.025	0.079
EEF1A1P5	0.099	0.049	0.043	8.858	7.268	0.000	0.076
C6orf62	0.097	0.049	0.047	11.060	8.161	0.026	0.000

**Table 2:** Evaluate the model with leave one out.

Model	AUC	CA	F1	Precision	Recall
SVM	0.447	0.710	0.626	0.560	0.710
Random Forest	0.784	0.761	0.687	0.726	0.761
Naive Bayes	0.914	0.870	0.873	0.880	0.870
Logistic Regression	0.660	0.732	0.677	0.665	0.732

**Table 3:** Classifier model testing.

Model	AUC	CA	F1	Precision	Recall
SVM	0.771	0.783	0.710	0.831	0.783
Random Forest	0.928	0.841	0.810	0.868	0.841
Naive Bayes	0.954	0.891	0.894	0.903	0.891
Logistic Regression	0.758	0.775	0.722	0.754	0.775

### Immune-Related miRNA-mRNA Regulatory Network in Diabetic Pancreatic Cancer Tumor Tissue

**Figure 7:**
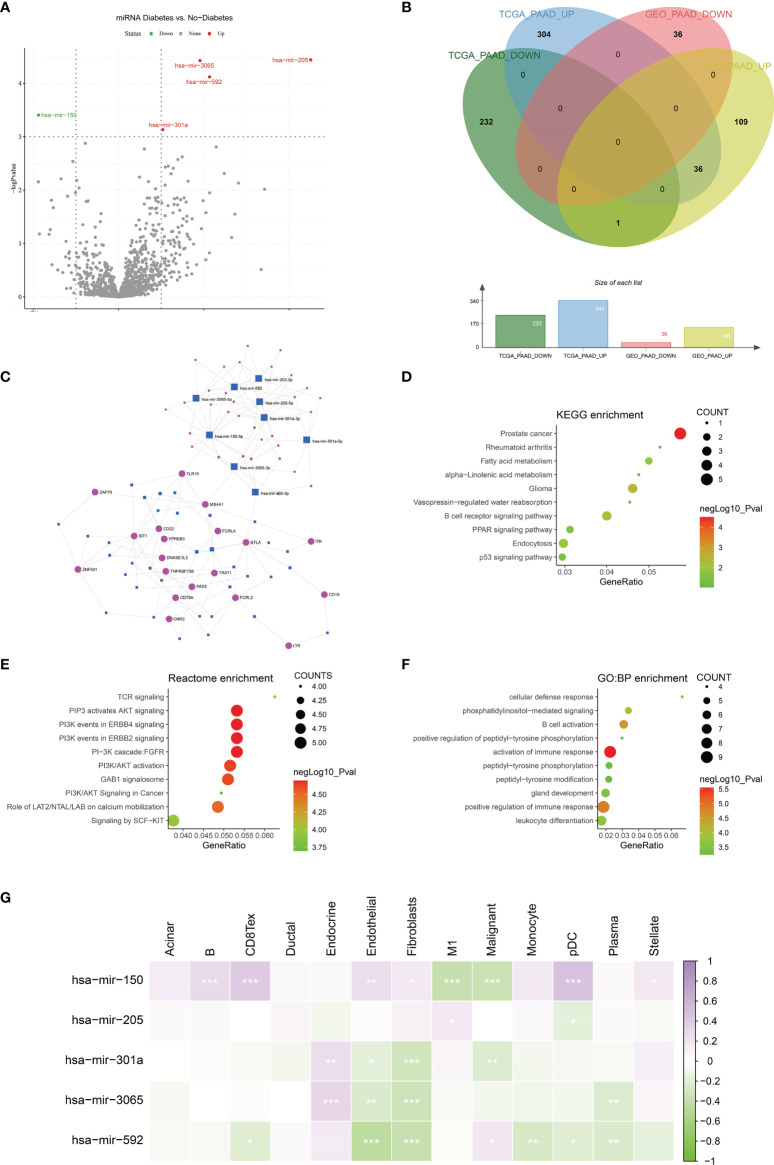
Construction of miRNA-mRNA regulatory network. **(A)** Differential expression analysis was performed on the Diabetes and no-diabetes subgroups of the TCGA PAAD miRNA dataset to find miRNAs that were significantly different between the two groups.**(B)** Screening of the intersection of differentially expressed genes from the TCGA PAAD dataset and the GSE79668 dataset. 36 genes that were highly expressed in pancreatic cancer diabetic patients were screened. genes were screened out. **(C)** Construction of miRNA-mRNA network interactions maps based on differentially expressed miRNAs and mRNAs. **(D–F)** Functional enrichment analysis of miRNA-mRNA regulatory networks. miRNA-mRNA networks are functionally enriched, mainly in immune and cancer-related signalling pathways. **(G)** The relationship between miRNAs and cellular infiltration in tumor microenvironment cells (Spearman correlation test, *p < 0.05, **p < 0.01, ***p < 0.001).

**Figure 8:**
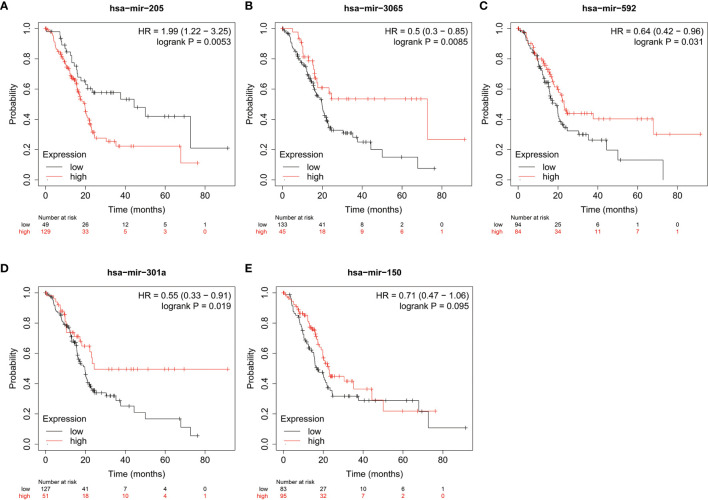
The relationship between miRNA and prognostic survival of PAAD patients. **(A–C)** High expression of hsa-mir-3065 (HR=0.50, p < 0.01), hsa-mir-592 (HR=0.64, p<0.05) and hsa-mir-301a (HR=0.55, p < 0.05) is a beneficial factor for PAAD. **(D)** High expression of hsa-mir-150 was a beneficial factor for PAAD, although not significant (HR=0.71, 0.05<p<0.1). **(E)** High expression of hsa-mir-205 is a high risk factor for PAAD (HR=1.99, p < 0.01).

MiRNAs can influence the regulation of gene expression by interfering with the expression of mRNAs. Given that diabetic pancreatic cancer has a specific “hot” immune state in the tumour microenvironment, the miRNA-mRNA regulatory network may be important for the maintenance of this immune state. TCGA PAAD miRNA expression profile data were used for differential expression analysis of diabetic pancreatic cancer versus non-diabetic pancreatic cancer (**Figure 7A**). Based on the filtering criteria of pvalue<0.001, |log2Foldchange|>0.5, we obtained four miRNAs upregulated in diabetic pancreatic cancer (hsa-mir-301a, hsa-mir-3065, hsa-mir-205, hsa-mir-592) and one downregulated miRNA (hsa-mir-150). By combining the results of TCGA PAAD and GSE79668, we found 36 genes upregulated in diabetic pancreatic cancer (**Figure 7B**). Using the miRNet, we constructed a miRNA-mRNA regulatory network (**Figure 7C**). TLR10, MS4A1, BTLA were the main nodes linking the miRNA regulatory module to the mRNA regulatory module. kEGG, Reactome, GO : BP databases were used for functional enrichment analysis of genes in the regulatory network, respectively. The results of the KEGG functional enrichment showed that this regulatory network is mainly associated with Prostate cancer, Rheumatoid arthritis, Fatty acid metabolism, etc(**Figure 7D**). The results of the functional enrichment of the Reactome database indicate that this regulatory network is mainly associated with TCR signaling, PIP3 activates AKT signaling, PI3K events in ERBB4 signaling, etc (**Figure 7E**). The results of the functional enrichment of the GO : BP database indicate that this regulatory network is mainly associated with cellular defense response, The results of functional enrichment in the GO : BP database suggest that this regulatory network is mainly associated with cellular defense response, phosphatidylinositol-mediated signaling, B cell activation and other signaling pathways (**Figure 7F**). To reveal the relationship between miRNAs and the tumour microenvironment, we calculated the spearman correlations of hsa-mir-150, hsa-mir205, hsa-mir-301a, hsa-mir-3065, hsa-mir-592 with 13 cell types (**Figure 7G**). The results showed that hsa-mir-150 positively correlated with B, CD8Tex, Endothelial, Fibroblasts, pDC, and Stellate cells (p<0.05), while negatively correlated with M1 and Malignant (p<0.05). This result suggests that hsa-mir-150 can promote the proliferation of cancer inflammation-related cells and inhibit cancer progression. Hsa-mir-301a was negatively correlated with Endothelial (p<0.05), Fibroblasts, and Malignant, and positively correlated with Endocrine (p<0.05). It suggests that hsa-mir-301a may promote the maintenance of Endocrine and inhibit cancer progression. Hsa-mir-3065 was positively correlated with Endocrine (p<0.05) and negatively correlated with Endothelial, Fibroblasts, and Plasma (p<0.05), this result suggests that hsa-mir-3065 may be important for maintaining Endocrine and inhibiting vascularization and fibrosis in tumor tissues.Hsa-mir-592 was negatively correlated with CD8Tex, Endothelial, Fibroblasts, Monocyte, pDC, and Plasma (p<0.05) and negatively correlated with Malignant (p>0.05), suggesting that its role in the tumour microenvironment may primarily be to inhibit tumour vascularisation and fibrosis and promote cancer progression. To demonstrate the important role of these miRNAs, the Kaplan-Meier Plotter was used to assess the relationship between these five miRNAs and prognostic survival of cancer patients. The results showed that high expression of hsa-mir-3065 (**Figure 8A**, HR=0.5, p<0.01),hsa-mir-592 (**Figure 8B**, HR=0.64, p<0.05), hsa-mir-301a (**Figure 8C**, HR=0.55, p<0.05),hsa-mir-150 (**Figure 8D**, HR=0.71, p=0.095) was a pancreatic cancer beneficial factor, while high expression of hsa-mir-205 was a risk factor for pancreatic cancer (**Figure 8E**, HR=1.99, p<0.01). These results demonstrate that we can modulate the miRNA-mRNA regulatory network through miRNAs, thereby altering the state of the tumour immune microenvironment in pancreatic cancer tissues and thereby improving the prognostic survival of pancreatic cancer patients.

## Discussion

Diabetes mellitus and pancreatic cancer are two interrelated diseases. The results of some studies suggest that diabetes has no significant effect on the duration of OS (5, 30). However, other studies have shown that diabetes significantly reduces OS (31, 32). A recent meta-analysis has shown that diabetes is associated with increased overall mortality in patients with pancreatic cancer and that patient survival also depends on the stage of the tumour and the duration of diabetes (33). These results suggest a complex mechanism for the presence of diabetes in patients with pancreatic cancer.

In this study, we explored differences in the tumour microenvironment between diabetic and non-diabetic pancreatic cancers. We studied two separate datasets with diabetic status in pancreatic cancer patients, from the TCGA-PAAD dataset and the GSE79668 dataset, respectively. After differential analysis, overexpression analysis and GSEA analysis, we found that immune and inflammation-related signalling pathways were activated in the tumour tissues of diabetic pancreatic cancer patients relative to the normal pancreatic cancer patients. The ‘‘hot’’ immune status of pancreatic cancer tumor tissue means higher immune cell infiltration and better prognosis for survival (34). The “hot” immune state in the tumor tissue of diabetic pancreatic cancer patients is most probably due to their diabetes-induced chronic inflammatory response (35). To further explore the composition of the pancreatic cancer tumour microenvironment at the cellular level, we constructed a matrix of immune cell infiltration characteristics of 13 pancreatic cancer microenvironments using single cell sequencing data to analyse the differences in the cellular composition of the tumour microenvironment in diabetic pancreatic cancer patients compared to non-diabetic pancreatic cancer patients. The results showed that CD8Tex scores were higher and Malignant cell scores were lower in diabetic pancreatic cancer compared to normal pancreatic cancer patients. Significant differences were found between the two subgroups. A significant negative correlation was shown between CD8Tex and Malignant. This suggests that immune infiltration of CD8Tex can significantly reduce the malignant progression of pancreatic cancer. It is in general accordance with the pathological section data from the TCGA-PAAD dataset (**Supplementary Figure S3**). We also found that the mean scores of Acinar cell, B cell and stellate cell were higher in diabetic pancreatic cancer, although less significant. The findings of the tumour microenvironment scores were consistent for both datasets. This non-significance may be caused by the small number of diabetic pancreatic cancer samples. CD8+ cytotoxic T cells are the main functional cells of cellular immunity, which can directly recognize tumour cells and secrete cytotoxic factors such as perforin and granzyme to kill tumour cells (36). A recent systematic appraisal and meta-analysis has shown that high tumour infiltration of T cells in pancreatic cancer promises better survival, and in particular that high infiltration of CD8+ T cells leads to better prognostic outcomes. T-cell infiltration located at the centre of the tumour has the greatest impact on cancer survival (37). Evidence from another study suggests that the spatial distribution of CD8+ T cells in the tumour microenvironment of pancreatic ductal carcinoma has an important impact on prognosis (38). From our results, patients with high CD8+ T cell immune infiltration had better prognostic survival. CD8+ T cells improve prognostic survival by killing malignant tumour cells and thus inhibiting their progression. Although the prognostic survival of diabetic pancreatic cancer patients with high infiltration of CD8+ T cells was not significantly improved in our study, it suggests that the two diseases, diabetes and pancreatic cancer, show a complex pattern of relationship in the tumour tissue of patients with diabetes combined with pancreatic cancer. Furthermore, we found that high infiltration of Malignant and M1 cells correlated highly with prognostic survival in pancreatic cancer. In the TCGA PAAD dataset, immune infiltration of M1 was a high risk factor for pancreatic cancer prognosis in both the diabetic and non-diabetic groups.

We estimated the functional status of T lymphocytes in pancreatic cancer diabetes versus non-pancreatic cancer diabetes using the TIDE computational framework. The results showed that there was no significant difference in the dysfunctional scores of T lymphocytes between the two subgroups, while the immune escape scores of T lymphocytes may have been lower in the diabetic pancreatic cancer group. These findings suggest that T lymphocytes in the tumour microenvironment of diabetic pancreatic cancer patients are not only more infiltrated than in non-diabetic patients, but may also be more functional.

In addition, we constructed a Naïve Bayes classifier using a machine learning approach. This classifier has excellent classification ability to distinguish patients with diabetic pancreatic cancer from those with non-diabetic pancreatic cancer. This classifier could be used to automatically classify pancreatic cancer RNA sequencing data in databases without diabetes status annotation, thereby expanding the sample size for pancreatic cancer diabetes studies. No research team has done anything related to this so far.

Diabetic pancreatic cancer is in a “hot” immune state, and miRNAs may be critical in maintaining such an immune state. Through differential expression analysis, we identified five significantly differentially expressed miRNAs from the TCGA PAAD miRNA expression profile dataset, including hsa-mir-301a, hsa-mir-3065, hsa-mir-205, hsa-mir-592 and hsa-mir-150. The results of the Kaplan-Miere survival analysis show that these genes are prognostically essential in pancreatic cancer.Hsa-mir-301a can promote pancreatic cancer progression by down-regulating the SMAD4 gene (39). Hsa-mir-3065 can affect the growth of melanoma cells through multiple antitumor effects. However, its relevance to pancreatic cancer has been relatively little studied (40). Hsa-mir-205 is a highly conserved miRNA whose regulated genes are mainly involved in tumourigenesis, progression, cellular value-added and epithelial-to-mesenchymal transition processes. miRNA-205 is a potential biologic drug for cancer therapy (41). Gemcitabine combined with miRNA-205 regimen shows promising results in patients with advanced pancreatic cancer (42). High expression of Hsa-mir-592 can promote the value-added migration of colon cancer (43), and mir-592 in serum can be used as an early diagnostic marker for colon cancer (44). Hsa-mir-150 can promote the progression of non-small cell lung cancer by targeting FOXO4 (45). hsa-mir-150 can act as a plasma marker (46) of pancreatic cancer progression and a prognostic marker (47). All these studies have demonstrated the importance of these five miRNAs in the diagnosis and treatment of cancer. *via* miRNet, we construct miRNA-mRNA regulatory networks for differentially expressed miRNAs and mRNAs. Functional enrichment analysis of the KEGG, Reactome, and GO : BP databases revealed that these genes are closely related to the inflammatory response. Three genes, TLR10, MS4A1, and BTLA, are the hub genes linking the miRNA regulatory module and the mRNA regulatory module. TLR10 is a member of a family encoding toll-like receptors (TLRs) that perform important roles in pathogen recognition and activation of the innate immune system (48). MS4A1, also known as CD20, encodes a B-lymphocyte surface molecule that plays a role in the development and differentiation of B cells into plasma cells. CD20-positive pancreatic cancers with a high degree of B-cell infiltration have a better prognostic survival rate (49). The protein encoded by BTLA (CD272) contains an immunoglobulin (Ig) structural domain and is a receptor that transmits inhibitory signals as well as suppressing immune responses. It has been shown that BTLA can be considered as a prognostic marker for pancreatic cancer (50). In the miRNA regulatory module, we found that the direct targets of action of these miRNAs include a large number of oncogene (e.g. TP53, PTEN, ERBB3, etc.). It illustrates the potential of these miRNAs as gene therapy agents. These results suggest that miRNAs can influence the state of the pancreatic cancer tumour microenvironment through the regulation of their target genes, thereby improving prognostic survival in pancreatic cancer.

In summary, we have discovered differences in the tumour microenvironment between diabetic and non-diabetic pancreatic cancers for the first time and compared the differences between the two at the genetic and cellular levels. The “hot” immune state of diabetic pancreatic cancer may contribute to the reduction of malignant cells in the pancreatic cancer tumour micro-environment, thus affecting the progression and prognosis of diabetic pancreatic cancer.

## Data Availability Statement

The datasets presented in this study can be found in online repositories. The names of the repository/repositories and accession number(s) can be found in the article/**Supplementary Material**.

## Author Contributions

ZY: Methodology, Software, Validation, Formal analysis, Investigation, Writing-Original Draft, Data Curation, Supervision. DLL: Writing- Review & Editing, Formal analysis, Investigation, Data Curation. DCL: Formal analysis, Investigation YZ: Software, Validation. YL: Visualization, Supervision. JZ: Visualization, Supervision, Methodology. JB: Writing- Review & Editing. XY: Data Curation, Supervision. JH: Methodology. LL: Conceptualization, Project administration, Visualization, Supervision, Methodology, Investigation Supervision. All authors contributed to the article and approved the submitted version.

## Funding

This work was supported by National Natural Science Foundation of China (81970717, 82000740 and 82170845), grants from the Key Research & Development Program of Jiangsu Province (BE2018742).

## Conflict of Interest

The authors declare that the research was conducted in the absence of any commercial or financial relationships that could be construed as a potential conflict of interest.

## Publisher’s Note

All claims expressed in this article are solely those of the authors and do not necessarily represent those of their affiliated organizations, or those of the publisher, the editors and the reviewers. Any product that may be evaluated in this article, or claim that may be made by its manufacturer, is not guaranteed or endorsed by the publisher.

## Supplementary Material

Supplementary Figure S1 KEGG, GEO analysis of upregulated genes in non-diabetic pancreatic cancer patients and GSEA enrichment analysis of patients. **(A–C)** GO functional enrichment, GSEA and gene regulatory network maps in the TCGA PAAD dataset, respectively. **(D–F)** KEGG functional enrichment, GSEA and gene expression regulatory network from TCGA PAAD dataset, respectively. **(G–I)** GO functional enrichment, GSEA and gene expression regulatory network from GSE79668 dataset, respectively.

Supplementary Figure S2 Relationship between tumor microenvironment components and prognostic survival of cancer patients in the GSE79668 dataset. **(A)** Relationship between scores of CD8Tex, Endocrine, M1, and Malignant and prognostic survival of patients. **(B)** Comparison of prognostic survival of patients grouped with high and low tumour immune scores of Malignant. The results showed some difference but not significant (0.05<p<0.1).

Supplementary Figure S3 Comparison of H&E staining of diabetic pancreatic cancer vs. non-diabetic pancreatic cancer tissues. Diabetic pancreatic cancer tissue sections(A: TCGA-IB-AAUR-01A-02-TSB,C:TCGA-IB-AAUP-01A-01-TSA,E:TCGA-Q3-A5QY-01A-01-TSA) and non-diabetic pancreatic cancer tissue sections (B:TCGA-HV-A7OP-01A-TS1,D: TCGA-2L-AAQJ-01A-TS1, F:TCGA-FB-AAPP-01A-01-TSA) obtained from the TCGA-PAAD dataset. The diabetic pancreatic cancer tissue section has a more pronounced lymphocytic infiltration, while the non-diabetic pancreatic cancer tissue has a more apparent deterioration of the cancerous tissue.

## Abbreviations

TCGA, The Cancer Genome Atlas; GEO, Gene Expression Omnibus; TIDE, Tumor Immune Dysfunction and Exclusion; GSEA, Gene Set Enrichment Analysis; PAAD, Pancreatic adenocarcinoma; TISCH, Tumor Immune Single-cell Hub.
