# Supplementary figures and images for: Immune Infiltration of CD8+ T Cells in Patients With Diabetic Pancreatic Cancer Reduces the Malignancy of Cancer Tissues: An In Silico Study
Source: Front Endocrinol (Lausanne). 2022 Jan 25;12:826667. doi: 10.3389/fendo.2021.826667 (PMC8821103; doi:10.3389/fendo.2021.826667)

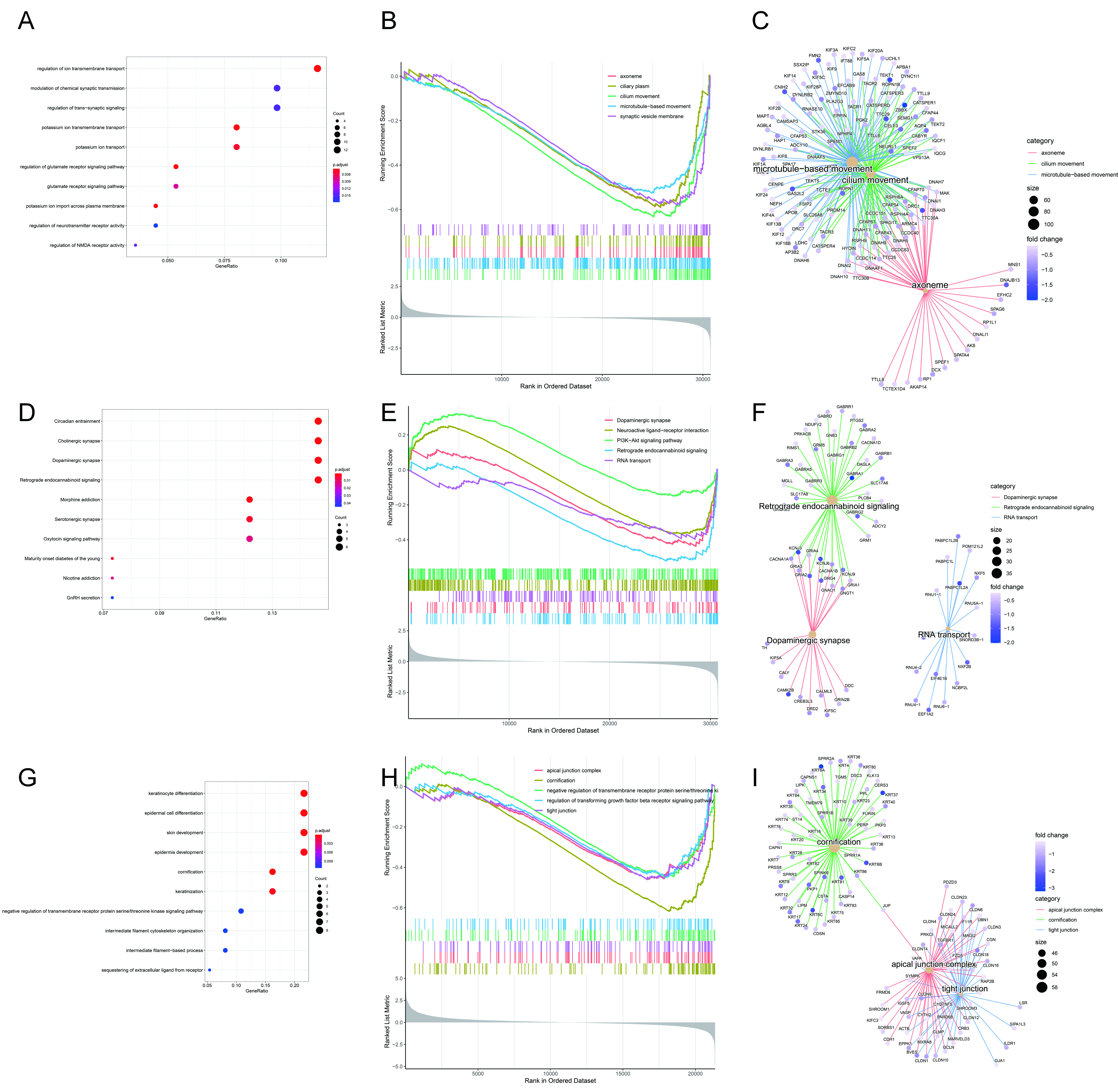

Supplement: Supplementary Figure S1 — KEGG, GEO analysis of upregulated genes in non-diabetic pancreatic cancer patients and GSEA enrichment analysis of patients. (A–C) GO functional enrichment, GSEA and gene regulatory network maps in the TCGA PAAD dataset, respectively. (D–F) KEGG functional enrichment, GSEA and gene expression regulatory network from TCGA PAAD dataset, respectively. (G–I) GO functional enrichment, GSEA and gene expression regulatory network from GSE79668 dataset, respectively. [file Image_1.jpeg]

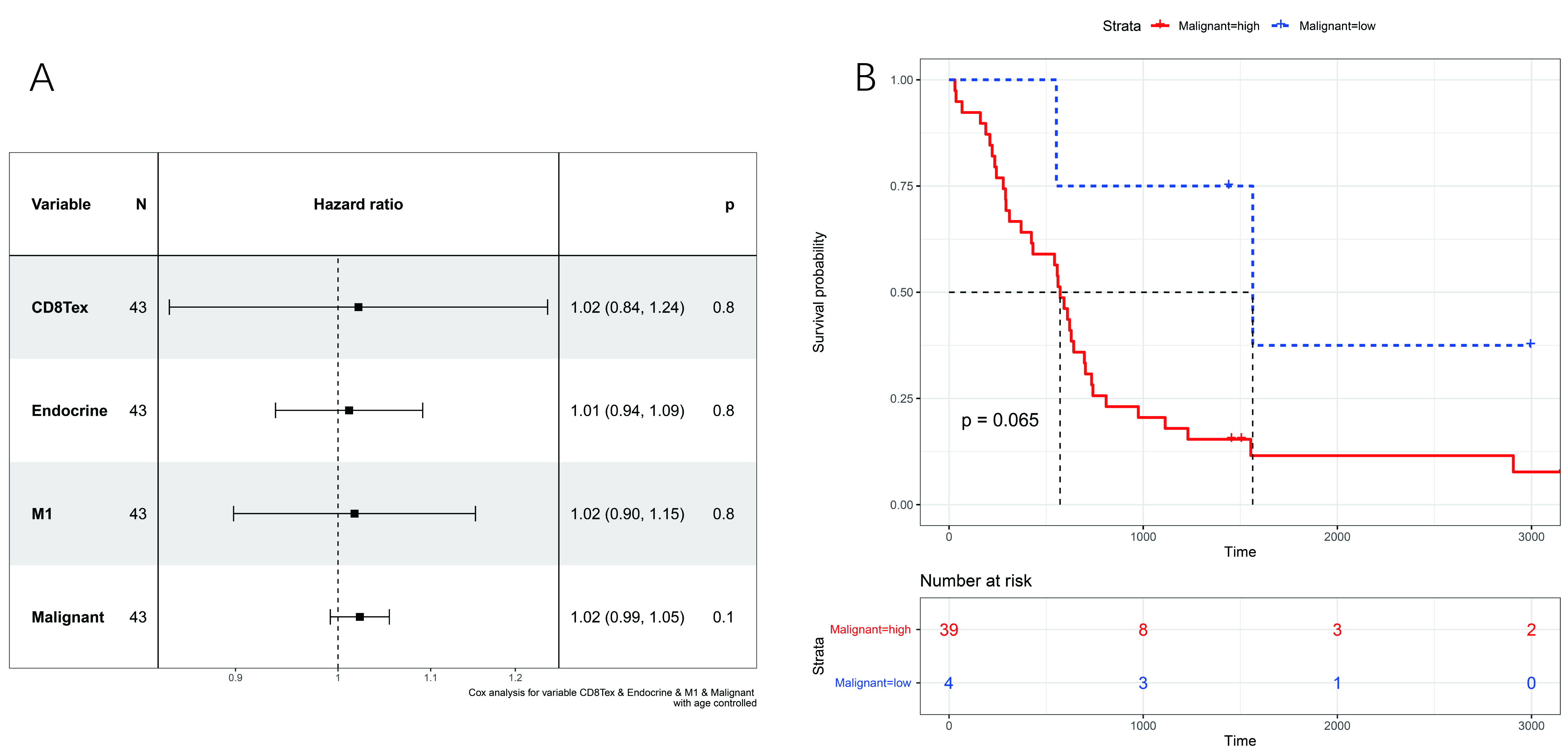

Supplement: Supplementary Figure S2 — Relationship between tumor microenvironment components and prognostic survival of cancer patients in the GSE79668 dataset. (A) Relationship between scores of CD8Tex, Endocrine, M1, and Malignant and prognostic survival of patients. (B) Comparison of prognostic survival of patients grouped with high and low tumour immune scores of Malignant. The results showed some difference but not significant (0.05<p<0.1). [file Image_2.jpeg]

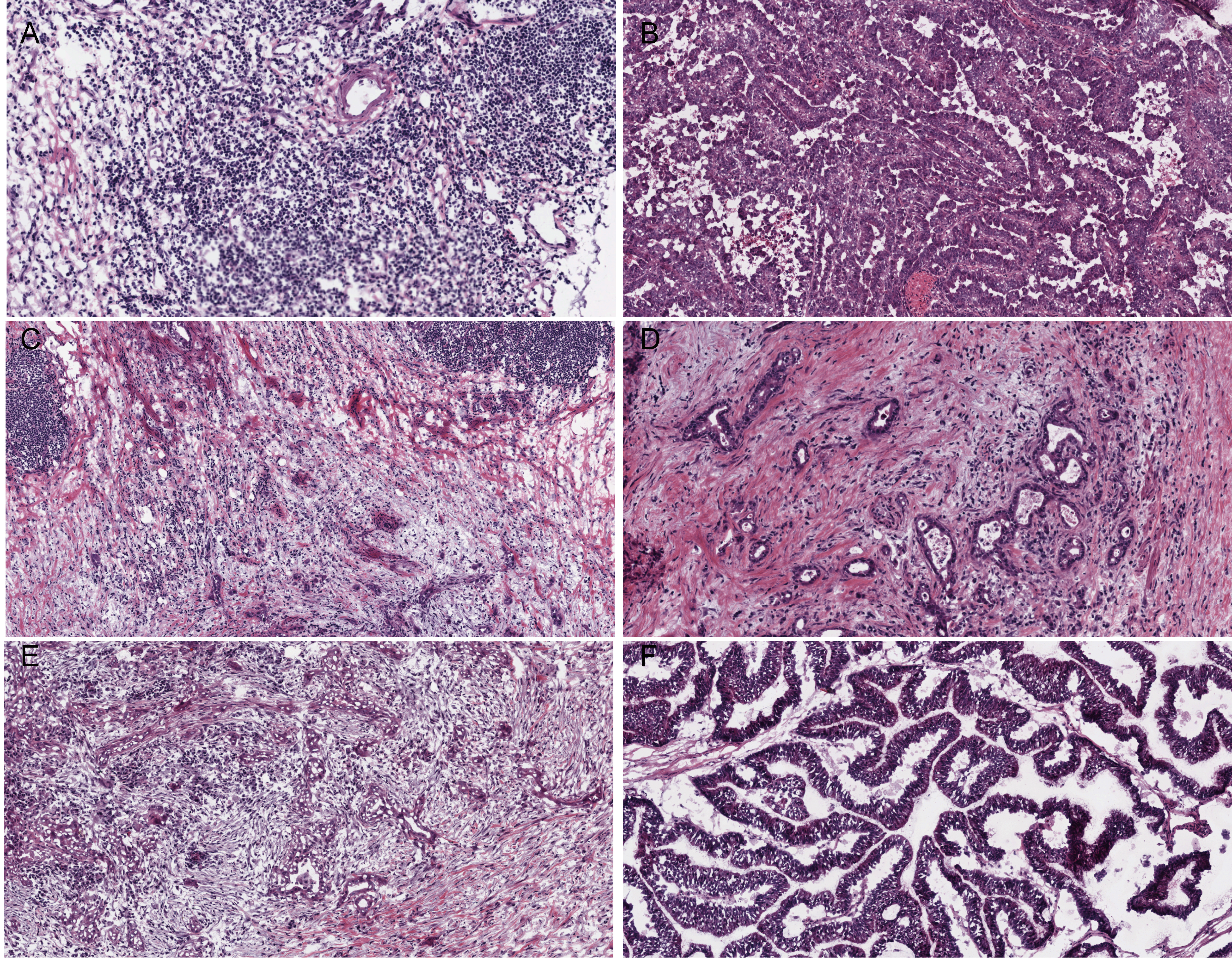

Supplement: Supplementary Figure S3 — Comparison of H&E staining of diabetic pancreatic cancer vs. non-diabetic pancreatic cancer tissues. Diabetic pancreatic cancer tissue sections(A: TCGA-IB-AAUR-01A-02-TSB,C:TCGA-IB-AAUP-01A-01-TSA,E:TCGA-Q3-A5QY-01A-01-TSA) and non-diabetic pancreatic cancer tissue sections (B:TCGA-HV-A7OP-01A-TS1,D: TCGA-2L-AAQJ-01A-TS1, F:TCGA-FB-AAPP-01A-01-TSA) obtained from the TCGA-PAAD dataset. The diabetic pancreatic cancer tissue section has a more pronounced lymphocytic infiltration, while the non-diabetic pancreatic cancer tissue has a more apparent deterioration of the cancerous tissue. [file Image_3.jpeg]

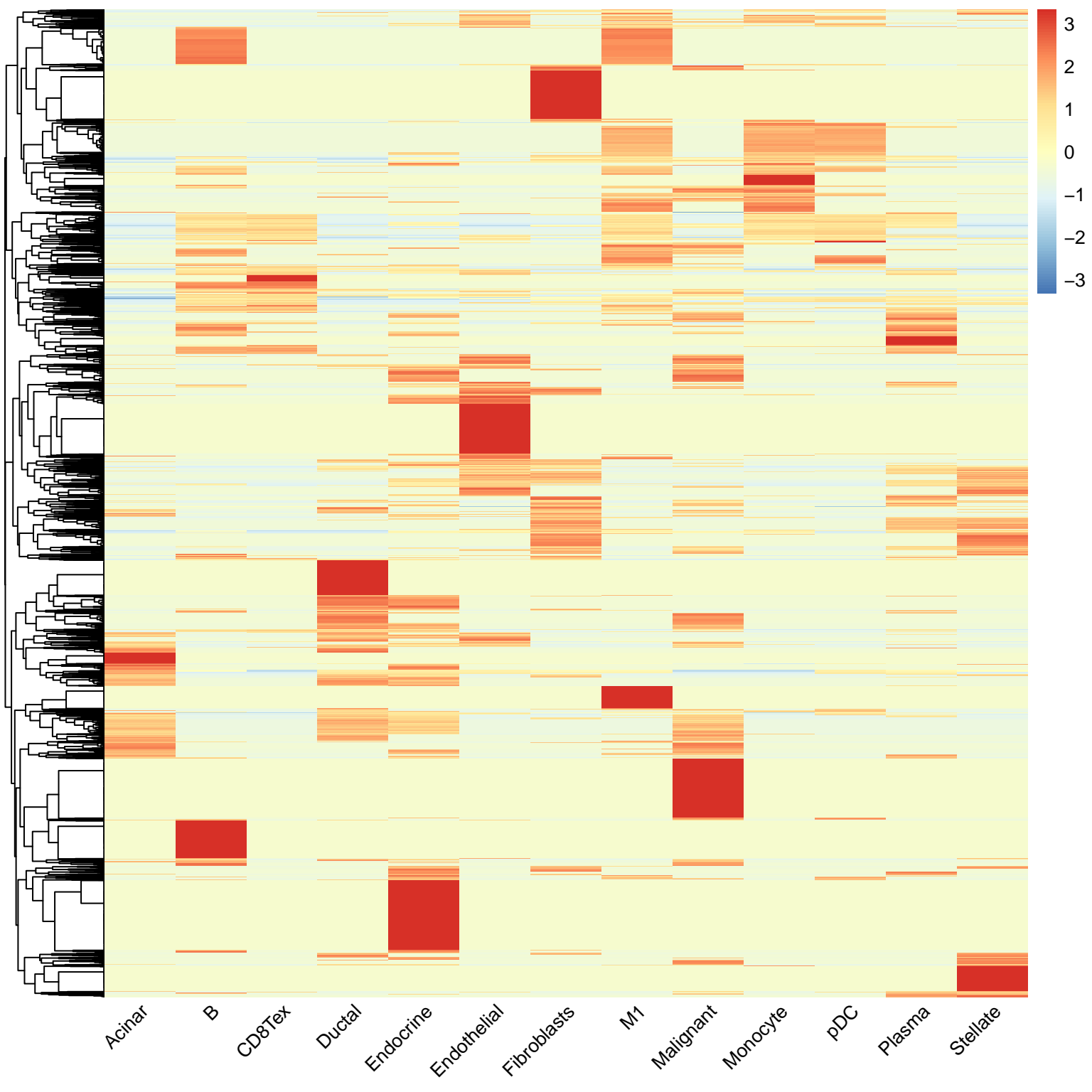

Supplement: Supplementary file 4 [file DataSheet_1.pdf]
